# Hollow MIL-125 Nanoparticles Loading Doxorubicin Prodrug and 3-Methyladenine for Reversal of Tumor Multidrug Resistance

**DOI:** 10.3390/jfb14110546

**Published:** 2023-11-13

**Authors:** Qingfeng Guo, Jie Li, Jing Mao, Weijun Chen, Meiyang Yang, Yang Yang, Yuming Hua, Lipeng Qiu

**Affiliations:** 1 Department of Thyroid and Breast Surgery, Affiliated Hospital of Jiangnan University, Wuxi 214122, China; guoqf_jdfy@outlook.com; 2 School of Life Sciences and Health Engineering, Jiangnan University, Wuxi 214122, China; 6211507007@stu.jiangnan.edu.cn (J.L.); 17851319532@163.com (J.M.); weijunchenaiwhl@outlook.com (W.C.); y13002666962@163.com (M.Y.); yangy@jiangnan.edu.cn (Y.Y.)

**Keywords:** hyaluronic acid, metal-organic framework, multidrug resistance, doxorubicin, 3-methyladenine

## Abstract

Multidrug resistance (MDR) is a key factor in chemotherapy failure and tumor recurrence. The inhibition of drug efflux and autophagy play important roles in MDR therapy. Herein, a multifunctional delivery system (HA-MIL-125@DVMA) was prepared for synergistically reverse tumor MDR. Tumor-targeted hollow MIL-125-Ti nanoparticles were used to load the doxorubicin–vitamin E succinate (DV) prodrug and 3-methyladenine (3-MA) to enhance reverse MDR effects. The pH-sensitive DV can kill tumor cells and inhibit P-gp-mediated drug efflux, and 3-MA can inhibit autophagy. HA-MIL-125@DVMA had uniformly distributed particle size and high drug-load content. The nanoparticles could effectively release the drugs into tumor microenvironment due to the rapid hydrazone bond-breaking under low pH conditions, resulting in a high cumulative release rate. In in vitro cellular experiments, the accumulation of HA-MIL-125@DVMA and HA-MIL-125@DV in MCF-7/ADR cells was significantly higher than that in the control groups. Moreover, the nanoparticles significantly inhibited drug efflux in the cells, ensuring the accumulation of the drugs in cell cytoplasm and causing drug-resistant cells’ death. Importantly, HA-MIL-125@DVMA effectively inhibited tumor growth without changes in body weight in tumor-bearing mice. In summary, the combination of the acid-sensitive prodrug DV and autophagy inhibitor 3-MA in a HA-MIL-125 nanocarrier can enhance the antitumor effect and reverse tumor MDR.

## 1. Introduction

Anticancer drugs, such as doxorubicin (DOX) and paclitaxel, are widely used to kill cancer cells in current clinical treatments, but cancer cells will develop a certain resistance to the drugs with the progression of time including a variety of anticancer drugs, which is called tumor multidrug resistance (MDR) [1,2,3,4]. MDR is a key factor in chemotherapy failure and tumor recurrence. The abnormal expression of ABC (ATP-binding cassette) [5,6,7] transporters is a significant cause of MDR produced by cancer cells, including P-glycoprotein (P-gp) [8,9,10], breast cancer drug resistance protein (BCRP) [11], multidrug resistance-associated protein (MRP) [12], etc. Among them, P-glycoprotein (P-gp) is the most representative member of the ABC transporter family, which is encoded by the MDR1 gene [13] and overexpressed in a variety of tumor-resistant strains. The two structurally transmembrane-binding domains of P-gp can bind to drugs, pump them out of the cells, and maintain low drug concentrations within the cell, resulting in MDR [14,15]. Many studies have shown that vitamin E and its derivative (such as vitamin E succinate (VES)) have safe and efficient anticancer activity and can reverse tumor MDR by inhibiting the activity of ATPase to prevent the efflux effect of P-gp against drugs in order to increase the accumulation of drugs in cells [16,17]. 

Nanocarriers have been widely studied in reversing tumor MDR [18,19]. They could effectively downregulate the expression of P-gp and enhance intracellular accumulation, leading to improve the therapeutic efficacy [20]. Among them, metal–organic framework nanoparticles (MOF NPs) are porous materials that have emerged in recent years. They are very regular porous skeleton structures formed by the self-assembly of metal ions and organic ligands [21,22], and have been widely used in catalysis [23], storage [24,25,26], adsorption [27,28,29], separation [30,31], biomedicine [32], etc. Compared with other drug carriers, MOF NP materials show irreplaceable advantages due to their large specific surface area, high porosity and easy modification [33,34,35]. For MDR, MOF NPs have been utilized to treat bacterial multidrug resistance induced by the abuse of traditional antibiotics [36], as well as cancer multidrug resistance caused by chemotherapy medicines [37,38]. Among the reported MOF NPs, MIL-125 MOF NPs, formed by a titanium ion group, are excellent representatives, but they lack active targeting and only rely on passive targeting by the enhanced permeability and retention effect (EPR effect) [39,40,41,42,43]. This affects the accumulation of drugs in the tumor site to a certain extent. Therefore, the introduction of special molecules on the surface of MIL-125 for chemical modification can achieve the active targeting of tumors [44].

In addition, autophagy is a method for cells to maintain the stability of their intracellular environment by decomposing deformed, damaged and non-functional proteins and cell structures via lysosomes to reduce nutritional stress when exposed to adverse situations such as starvation and hypoxia [45,46]. Autophagy assists tumor cells in rapidly adapting to external influences, increasing the cells resistance to treatment, and plays a crucial role in the process of MDR [47]. The inhibition of autophagy can significantly increase drug sensitivity and induce the apoptosis of tumor cells [48]. 3-methyladenine (3-MA) acts on the PI3K/Akt/mTOR signaling pathway by inhibiting the activity of PI3K, which can effectively inhibit autophagy and synergistically reverse tumor MDR [49,50]. In a previous studies, we prepared hyaluronic acid (HA)-modified MOF NPs (HA-MIL-125) for efficient drug delivery [51] and a DOX-VES (DV) prodrug containing a pH-sensitive hydrazone bond for tumor MDR therapy [52]. Herein, HA-MIL-125 NPs were used as nanocarriers to coload DV and 3-MA (HA-MIL-125@DVMA) to synergistically reverse tumor MDR by inhibiting P-gp efflux and autophagy. The pharmaceutical properties, in vitro cytotoxicity and uptake, drug efflux and in vivo antitumor effects were investigated to verify the MDR reversal effect. The excellent reversal effect of MDR makes HA-MIL-125@DVMA a promising nanomedicine for cancer therapy, and this study might offer a new strategy by the combination of the inhibition of autophagy and MOF NPs to jointly improve the effect of reversing MDR. 

## 2. Experimental Section

### 2.1. Materials

3-methyladenine (3-MA, ≥99%), ethyl acetate (≥99.5%), cyclohexane (≥99.5%), dichloromethane (≥99.5%) and anhydrous sodium sulfate were all purchased from Sinopharm Chemical Reagent. Vitamin E succinate (VES), tert-butyl carbazate and trifluoroacetic acid (TFA) were ordered from Sigma-Aldrich (St. Louis, MI, USA). The reagent 1-(3-dimethylaminopropyl)-3-ethylcarbodiimide hydrochloride (EDC) was provided from Shanghai Aladdin Bio-Chem Technology Co., Ltd. (Shanghai, China). Hyaluronic acid (HA, Mw: 10,000 Da, ≥99%) was ordered from Bloomage Biotechnology Corporation Limited (Jinan, China). Doxorubicin hydrochloride (DOX) was purchased from Dalian Meilune Biotech Co. Ltd. (Dalian, China). All reagents were used as received without further purification.

### 2.2. Cell Cultures and Animal Models

Human breast carcinoma cells (MCF-7) and Human breast cancer doxorubicin-resistant cells (MCF-7/ADR) were provided by Chinese Academy of Sciences (Shanghai, China) and Shanghai Gefan Biotechnology Co., Ltd. (Shanghai, China), respectively. MCF-7 cells were cultured in DMEM with 10% of FBS, 100 mg/mL streptomycin sulfate and 100 U/mL penicillin G sodium. MCF-7/ADR cells were maintained in DMEM supplemented with 10% of FBS, 100 mg/mL streptomycin sulfate, 100 U/mL penicillin G sodium and 500 ng/mL doxorubicin hydrochloride. Female BALB/c nude mice (6 weeks of age) were obtained from Shanghai SLAC laboratory animal Co., Ltd. (Shanghai, China). All animal procedures were carried out following the protocol approved by the Animal Study Committee of Jiangnan University (JN. No20190315n0720515[23]).

### 2.3. Preparation of HA-MIL-125@DVMA

DV was synthesized following the method previously reported [52]. The preparation method of co-encapsulated DV and 3-MA nanoparticles was as follows: 20.0 mg DV and 20.0 mg 3-MA were dissolved in 10 mL anhydrous N,N′-dimethylformamide (DMF), in which 30 mg of HA-MIL-125 was dispersed. The mixture was stirred for 24 h at room temperature, protected from light, centrifuged (10,000 r/min, 10 min), and washed with deionized water three times to remove the residual organic solvent. Finally, drug-loaded MOF nanoparticles (named HA-MIL-125@DVMA) were obtained. The control groups consisting of HA-MIL-125@DV and HA-MIL-125@DOX were prepared with the same method.

### 2.4. Characterization of the Nanoparticles

The average particle size, polydispersity coefficient (PDI) and zeta potential of HA-MIL-125@DVMA, HA-MIL-125@DV and HA-MIL-125@DOX were measured by dynamic light scattering (DLS, Zetasizer Nano ZS apparatus, Malvern, UK). Their morphology was observed using a scanning electron microscope (SEM, SU1510, Hitachi, Japan) and transmission electron microscope (TEM, JEM-2100, JEOL, Tokyo, Japan).

### 2.5. Determination of Drug Loading Content

The standard curve of DOX was determined using a ultraviolet and visible spectrophotometer (UV-vis, UV-2550, Shimadzu, Kyoto, Japan) at 479 nm. The drug-loading content (DC) of the nanoparticles was also determined by UV-vis. DOX and HA-MIL-125 were dissolved in DMF, and the solution was then added into water under sonication. The obtained solution was dialyzed against water until the water outside of the dialysis tube exhibited the negligible UV absorption of DOX. The drug-loaded nanoparticles were obtained by the overnight lyophilization of the dialysate. The formula for calculating the drug loading of the drug-carrying nanoparticles is as follows:

(1) $$DC\left(\%\right)=\frac{We}{We+Wm}\times 100$$

where We is the amount of DOX encapsulated in nanoparticles, and Wm is the amount of the nanoparticles.

### 2.6. In Vitro Drug Release

The in vitro drug release was investigated by dialysis. 2.0 mL of DOX-containing nanoparticles was added to the dialysis bag (MWCO: 3500 Da) that was placed in 20 mL PBS solution (pH 5.0 or 7.4, 0.1 M) and kept in a shaker at 37 °C, 100 rpm/min. 1.0 mL of sample was extracted at a specific time point, and the same volume of fresh PBS was supplemented to maintain the release system. The concentration of DOX was determined and calculated as follows:

(2) $$Er\left(\%\right)=\frac{V_{0}C_{n}+V_{e}\sum_{1}^{n-1}C_{i}}{m_{DOX}}\times 100$$

where Er(%) denotes the drug’s cumulative release, V_0_ is the total volume of PBS, C_n_ is the DOX concentration at the nth sampling, V_e_ is the volume of each sample, and m_DOX_ is the entire mass of DOX contained in the nanoparticle.

### 2.7. In Vitro Cytotoxicity Tests

In this experiment, the cytotoxicity of the blank and drug-loaded nanoparticles was detected using MCF-7 and MCF-7/ADR cells. Cells were seeded in 96-well plates at a density of 6 × 10^3^ cells/well, allowing cells to adhere and grow for 24 h. Then, the old medium was removed with a pipette gun, and new medium containing different concentrations of blank nanocarriers and drug-loaded nanoparticles were added. After incubation for 48 h, 100 L of an MTT solution (0.5 mg/mL) was added to each well for 4 h. Finally, DMSO was added to measure the absorbance at 570 nm to determine cell viability and IC_50_ values.

(3) $$Cell\;viability\left(\%\right)=\frac{E_{g}}{C_{g}}\times 100$$

where E_g_ and C_g_ represent the absorbance with and without sample treatment, respectively.

### 2.8. In Vitro Cellular Uptake Assays

#### 2.8.1. Qualitative Observation

The uptake of HA-MIL-125@DVMA, HA-MIL-125@DV, HA-MIL-125@DOX and free DOX by MCF-7 and MCF-7/ADR cells was qualitatively observed by confocal laser scanning microscopy (CLSM, ECLIPSE Ti2, Nikon, Tokyo, Japan). The cells were incubated in laser confocal dishes at a density of 1 × 10^5^ cells/dish for 24 h. 2 mL of medium containing various formulations (5.0 μg/mL of DOX) was incubated for different times. Then, the cells were washed with PBS at pH 7.4 and fixed with 200 μL of 4% paraformaldehyde solution for 15 min, followed by staining with 200 μL DAPI for 20 min for nuclei visualization. Finally, they were washed three times with PBS to remove the residual DAPI and 300 μL of PBS was added to each dish for observation by CLSM.

#### 2.8.2. Quantitative Analysis by Flow Cytometry

MCF-7 and MCF-7/ADR cells were seeded at a density of 4 × 10^5^ cells/well in 6-well plates and cultured for 24 h to grow adherently. The old medium was removed with a pipette, and 2 mL of new medium containing HA-MIL-125@DVMA, HA-MIL-125@DV, HA-MIL-125@DOX and free DOX (5.0 μg/mL of DOX) was added for incubation at different times. After incubation, the cells were washed with PBS and collected by centrifugation (3000 rpm, 5 min) for analysis by flow cytometry (Becton Dickinson FACSCalibur, Franklin Lakes, NJ, USA).

### 2.9. Drug Uptake and Efflux Experiments

The uptake and efflux of drug-loaded nanoparticles and free DOX by MCF-7/ADR cells were quantitatively determined by flow cytometry. MCF-7/ADR cells were seeded in a six-well plate at a density of 1 × 10^5^ cells/well and allowed to develop naturally for 24 h. Then, each dish was treated with 2 mL of media containing different formulations (5.0 μg/mL of DOX) for 12 h. After that, the incubation process was then continued with drug-free media for an additional 4 h. Lastly, the cells were washed by cold PBS three times, and 0.5 mL PBS was used to suspend the cells before the flow cytometry test.

### 2.10. In Vivo Antitumor Activity Studies

A tumor-bearing animal model was established by the subcutaneous injection of 100 μL of serum-free medium containing 1 × 10^7^ MCF-7/ADR cells into the abdomen. Subsequently, the volume of the tumor was measured every day, and when the tumor volume grew to 50~100 mm^3^, the administration was started. The mice were divided into 4 groups: control, free DOX, HA-MIL-125@DV and HA-MIL-125@DVMA. On day 1, 3, 5 and 7, mice were injected with the corresponding preparation (10 mg/kg, 200 μL/pcs) by tail vein, and normal saline was used as the control. After administration, the animals’ weight as well as tumor volume were recorded every day. The mice were sacrificed on day 10 following administration, and subcutaneous tumors were isolated and weighed. The tumor volume (mm^3^) was calculated as follows:

(4) $$Tumor\;volume=\frac{LW^{2}}{2}$$

where *L* and W are tumor’s long diameter and short diameter, respectively.

### 2.11. Pathological Examination

Hematoxylin and eosin (H&E) staining was adopted to further evaluate antitumor effects and toxicity of the nanoparticles in various organs. The samples were fixed with a 4% formaldehyde solution, stained and cut into 10 μm of slices. Finally, all specimens were observed by brightfield microscopy (IX73, Olympus, Tokyo, Japan).

### 2.12. Statistical Analysis

The results obtained were analyzed using a one-tailed Student’s *t*-test (SPSS, Chicago, IL, USA). All data are presented as the mean ± standard deviation (SD), and *p* values <0.05 were considered statistically significant.

## 3. Results and Discussion

### 3.1. Characterization of the Nanoparticles

**Figure 1:**
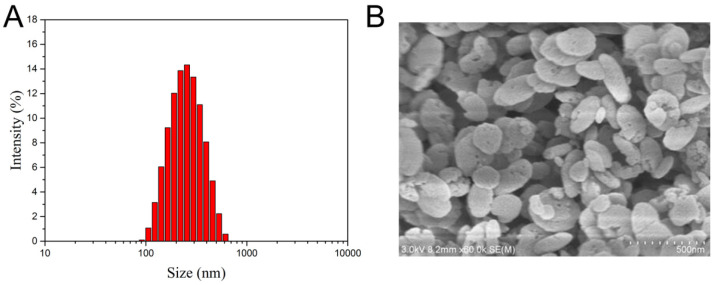
Size distribution (**A**) and SEM image (**B**) of HA-MIL-125@DVMA NPs.

The particle size and zeta potential of the drug-loaded nanoparticles are displayed in Table 1. The particle size of all nanoparticles was uniform and around 200 nm with negative charges. HA-MIL-125@DVMA showed a narrow range of size distribution (Figure 1A), and flat and spherical morphology (Figure 1B). In addition, HA-MIL-125@DVMA, HA-MIL-125@DV and HA-MIL-125@DOX all showed a high drug-loading content (Table 1), and it was speculated that the interaction between the drugs and HA-MIL-125 promoted the drug-loading effect.

### 3.2. In Vitro Drug Release

**Figure 2:**
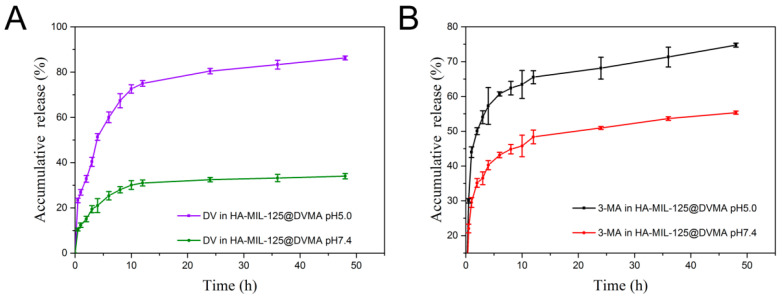
In vitro drug-release profiles of (**A**) DV and (**B**) 3-MA from HA-MIL-125@DVMA at pH 5.0 and pH 7.4. The data are presented as the mean ± SD, *n* = 3.

The drug release behavior of HA-MIL-125@DVMA was studied by mimicking the tumor lysosomal environment (pH 5.0) and normal physiological environment (pH 7.4), respectively. As shown in Figure 2, both DV and 3-MA showed a significant pH-sensitive release behavior. As for DOX released from DV, the total drug-release amount was about 35% (0.42 mg) at pH 7.4 at 48 h, while it was more than 85% (1 mg) at pH 5.0, which is significant difference (*p* < 0.001). This might be because the hydrazone bond in DV is pH-sensitive and can rapidly dissolve in acidic environment to release DOX. Additionally, the cumulative release of 3-MA at pH 5.0 and pH 7.4 was 74.73% (0.89 mg) and 55.35% (0.66 mg) at 48 h, respectively. This showed that the nanoparticles can effectively release 3-MA in tumor cells to assist in reversing MDR. In summary, HA-MIL-125@DVMA can rapidly release the drugs in the tumor microenvironment, which has a synergistic effect.

### 3.3. In Vitro Cytotoxicity

**Figure 3:**
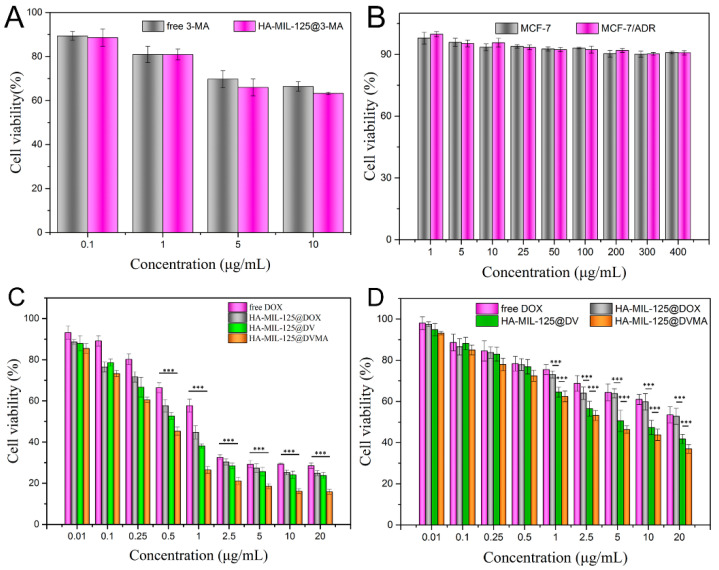
(**A**) Screening of 3-MA concentration using MCF-7/ADR cells. (**B**) The cytotoxicity of blank HA-MIL-125 nanoparticles against MCF-7 and MCF-7/ADR cells. The cytotoxicity of free DOX and drug-loaded NPs against (**C**) MCF-7 and (**D**) MCF-7/ADR cells. The data are presented as the mean ± SD, *n* = 3, (***) *p* < 0.001.

The loading concentration of 3-MA was first screened by its cytotoxicity in MCF-7/ADR cells. As shown in Figure 3A, the MCF-7/ADR cell survival rate decreased as the concentration of 3-MA increased. But when the concentration was higher than 5 g/mL, the falling rate tended to be flat. Thus, 5 g/mL was chosen for 3-MA loading.

The cytotoxicity of blank HA-MIL-125 and drug-loaded nanoparticles in MCF-7 and MCF-7/ADR cells was also determined by the MTT method. As shown in Figure 3B, the cell viability of blank HA-MIL-125 nanoparticles in both cells exceeded 90% even at high concentrations (400 g/mL), demonstrating the low toxicity and good safety of HA-MIL-125. The cytotoxicity results of drug-loaded nanoparticles and free DOX were tested in MCF-7 cells. As shown in Figure 3C, the cytotoxicity of HA-MIL-125@DVMA, HA-MIL-125@DV and HA-MIL-125@DOX was significantly higher than that of free DOX (*p* < 0.001). The reason might be that HA modified on the nanoparticle surface exerts a good active targeting effect, which is specifically recognized, binds to CD44 receptors and then is taken up into the cells. However, the cytotoxicity of HA-MIL-125@DVMA (IC50 value of 0.36 ± 0.03 μg/mL) and HA-MIL-125@DV (IC50 value of 0.72 ± 0.05 μg/mL) was stronger than that of HA-MIL-125@DOX (IC50 value of 0.91 ± 0.08 μg/mL) because DV can be rapidly broken down under acidic conditions in the tumor to release DOX and VES, resulting in the synergistic killing of tumor cells. Furthermore, in MCF-7/ADR cells (Figure 3D), although HA-MIL-125@DOX was more cytotoxic than free DOX (*p* < 0.001), the IC50 values of both were high (22.36 ± 1.07 μg/mL and 25.93 ± 0.95 μg/mL, respectively), indicating that MCF-7/ADR cells were highly resistant to DOX, even if the drug was encapsulated in nanoparticles. However, the cytotoxicity of HA-MIL-125@DVMA (IC50 value of 4.46 ± 0.37 μg/mL) and HA-MIL-125@DV (IC50 value of 6.65 ± 0.51 μg/mL) was significantly greater than that of free DOX and HA-MIL-125@DOX *p* < 0.001). In addition to the active uptake of the nanoparticles into the cell, the released VES in cytoplasm affected the efflux function of P-gp, so DOX accumulated rapidly in the cell, resulting in the reversal of tumor MDR. Obviously, HA-MIL-125@DVMA had the most obvious effect of tumor MDR reversal (*p* < 0.001) because 3-MA can inhibit the autophagy of tumor cells, exerting the synergistic reversal of tumor MDR.

### 3.4. In Vitro Cellular Uptake

#### 3.4.1. CLSM Observation

**Figure 4:**
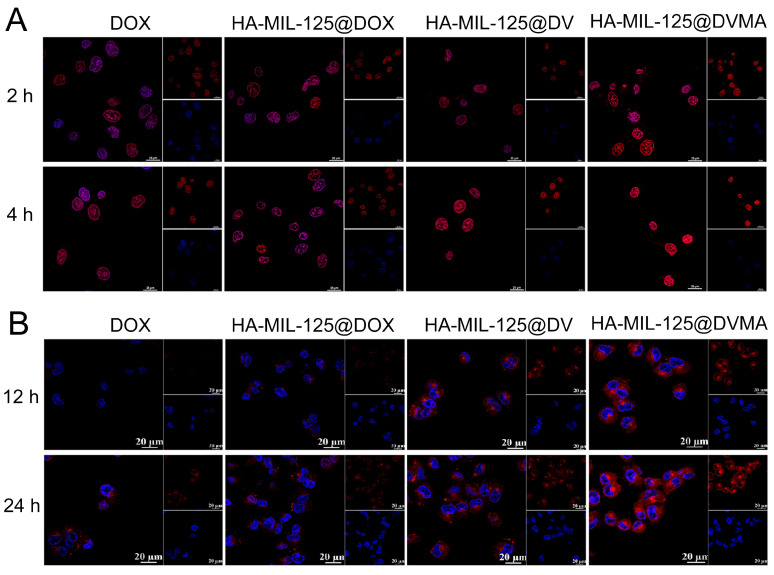
CLSM images of (**A**) MCF-7 cells and (**B**) MCF-7/ADR cells incubated with HA-MIL-125@DVMA, HA-MIL-125@DV, HA-MIL-125@DOX and free DOX at different times, showing DOX (red) and DAPI-stained nucleus (blue). The scale bar is 20 μm.

The internalization of HA-MIL-125@DVMA, HA-MIL-125@DV, HA-MIL-125@DOX and free DOX in MCF-7 and MCF-7/ADR cells was observed by CLSM. As shown in Figure 4A, the fluorescence intensity of all groups gradually increased with the extension of time in MCF-7 cells. At 2 h, free DOX was basically concentrated in the cytoplasm and nucleus because of the passive diffusion effect. It was worth noting that although the endocytosis of the nanoparticles and the hydrolysis of the prodrug required a certain amount of time, the fluorescence intensity of the drug-loaded nanoparticle groups was stronger than that of the free DOX group, which was mainly attributed to the active targeting function of HA. At 4 h, most of the DOX in each group was concentrated in the nucleus region, and the fluorescence intensity of HA-MIL-125@DVMA was the strongest, which was consistent with the cytotoxicity results. 

The cellular uptake of MCF-7/ADR cells at 12 h and 24 h is shown in Figure 4B. The DOX of each group was mainly distributed in the cytoplasm at 12 h, but the fluorescence intensity of the free DOX group was the weakest, resulting from the P-gp efflux pump. Although HA-MIL-125@DOX can actively bind to CD44 receptors to be taken up into the cells to avoid efflux, HA-MIL-125@DVMA and HA-MIL-125@DV, both containing VES, presented the highest fluorescence intensity. The hydrolysis of the DV prodrug produced VES, which protected DOX from being pushed out by the P-gp efflux. Therefore, the fluorescence intensities of the HA-MIL-125@DVMA and HA-MIL-125@DV groups were further strengthened at 24 h and gathered around the nuclei. Of course, HA-MIL-125@DVMA had the strongest fluorescence intensity at different times because of the addition of 3-MA. With the long-term use of chemotherapy drugs, the intracellular proteins in tumor cells are prone to misfolding, but autophagy can effectively remove the wrong proteins and provide raw materials for its biological processes to promote the development of drug resistance in cells. 3-MA can effectively reverse tumor MDR by inhibiting autophagy and increase the intracellular accumulation of chemotherapeutic drugs. 

#### 3.4.2. Flow Cytometry Test

**Figure 5:**
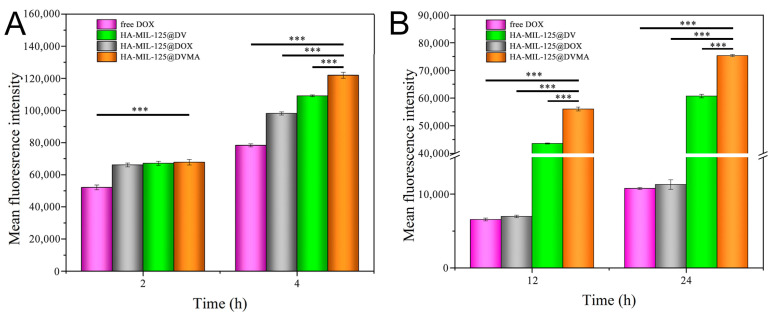
The fluorescence intensities of drug-loaded nanoparticles and free DOX in (**A**) MCF-7 and (**B**) MCF-7/ADR cells at different times. The data are presented as the mean ± SD, *n* = 3, (***) *p* < 0.001.

The cellular uptakes of different formulations by MCF-7 and MCF-7/ADR cells were quantitatively detected by flow cytometry. As shown in Figure 5, the uptake of DOX in both cells was a time dependent pattern. The uptake of drug-loaded nanoparticles in MCF-7 cells was significantly higher than that of free DOX, which resulted from CD44 receptor mediated endocytosis. It was worth noting that the cellular uptake of HA-MIL-125@DVMA was significantly higher than that of HA-MIL-125@DV (*p* < 0.001), which was consistent with the above results, indicating that 3-MA also had certain antitumor effect on non-drug-resistant tumor cells. Furthermore, the cellular uptakes of HA-MIL-125@DVMA and HA-MIL-125@DV nanoparticles were significantly higher than that of free DOX and HA-MIL-125@DOX at 12 h or 24 h, and the average fluorescence intensity of HA-MIL-125@DVMA nanoparticles was significantly stronger than that of HA-MIL-125@DV nanoparticles (*p* < 0.001). The result suggested that the efflux pump can pump DOX out of the cell, and VES which was released from pH-sensitive prodrugs can block P-gp effect for increasing the intracellular accumulation of DOX, meanwhile, 3-MA efficiently and synergistically reversed tumor MDR by inhibiting tumor autophagy.

### 3.5. Drug Efflux Assay

**Figure 6:**
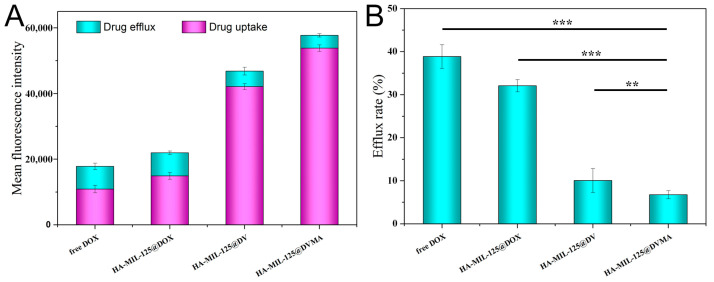
(**A**) The uptake and efflux and (**B**) efflux rate of drug-loaded nanoparticles and free DOX in MCF-7/ADR cells. The data are presented as the mean ± SD, *n* = 3, (**) *p* < 0.01, (***) *p* < 0.001.

The uptake and efflux of HA-MIL-125@DVMA, HA-MIL-125@DV, HA-MIL-125@DOX, and free DOX in MCF-7/ADR cells were quantitatively detected by flow cytometry. As shown in Figure 6, HA-MIL-125@DVMA and HA-MIL-125@DV nanoparticles significantly reduced efflux after uptake compared to HA-MIL-125@DOX and free DOX (*p* < 0.001). Obviously, the efflux rate of free DOX was about 40%, while HA-MIL-125@DVMA had the lowest efflux rate, with no more than 7% (*p* < 0.001). Moreover, the efflux rate of HA-MIL-125@DV was about 11%, which was higher than MIL-125@DVMA (*p* < 0.01). It can be inferred that the combination of DV and 3-MA can effectively prevent drug efflux for the synergistic reversal of tumor MDR.

### 3.6. In Vivo Antitumor Activity

**Figure 7:**
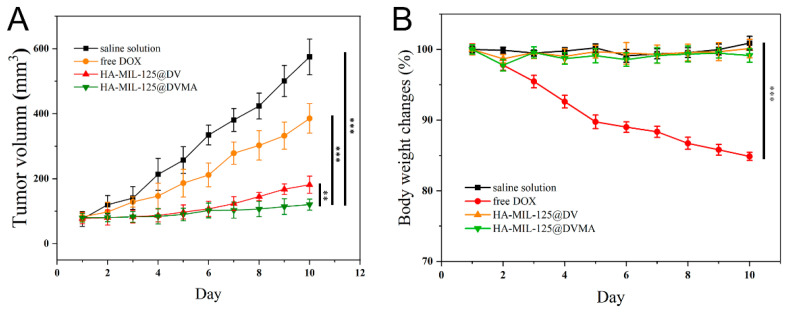
In vivo antitumor effect of different formulations. (**A**) Tumor volume and (**B**) body-weight changes of saline solution, free DOX, HA-MIL-125@DV and HA-MIL-125@DVMA. The data are presented as the mean ± SD, *n* = 5, (**) *p* < 0.01, (***) *p* < 0.001.

The tumor-targeting and antitumor effect of drug-loaded nanoparticles were evaluated by in vivo MDR tumor-bearing experiments. The antitumor effect was reflected by the changes in body weight and tumor volume. The tumor volume results from Figure 7A showed that the normal saline solution increased rapidly, followed by the free DOX group, while the tumor volume grew slowly in drug-loaded nanoparticle groups (*p* < 0.001), indicating that the drug-loaded nanoparticles can significantly inhibit tumor growth. Among them, HA-MIL-125@DVMA had the strongest inhibitory effect on tumors (*p* < 0.01), because 3-MA and the pH-sensitive DV prodrug exerted an efficient synergistic antitumor effect. Moreover, as shown in Figure 7B, the body weight of mice in the free DOX group decreased significantly (*p* < 0.001), while there was no significant difference in the drug-loaded nanoparticle groups and normal saline solution (*p* > 0.05). These results showed that HA-MIL-125 as nanocarriers for delivery of 3-MA and the DV prodrug can not only effectively reverse tumor MDR but also alleviate the toxic side effects of DOX.

**Figure 8:**
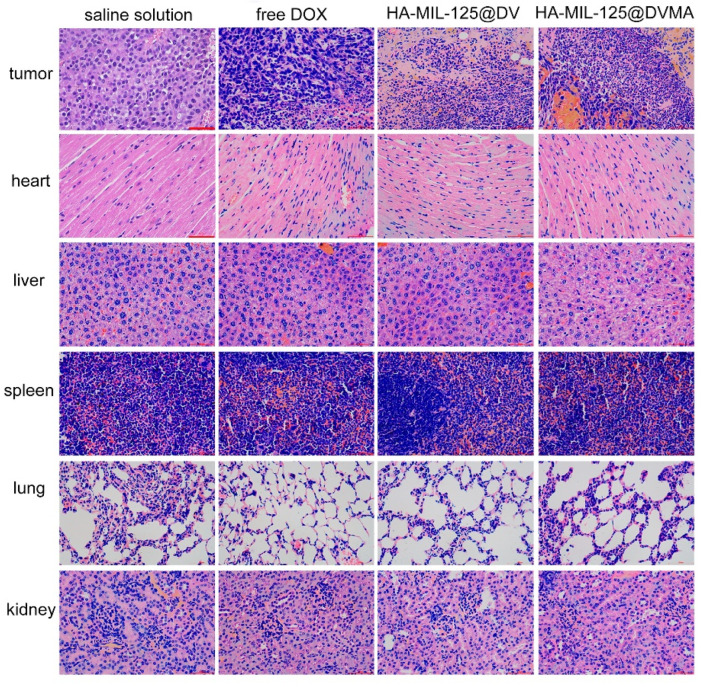
H&E staining images of different organs and tumors for saline solution, free DOX, HA-MIL-125@DV and HA-MIL-125@DVMA. The scale is 50 μm.

Furthermore, the toxicity of HA-MIL-125@DVMA on various organs was evaluated by hematoxylin and eosin (H&E) staining. As shown in Figure 8, HA-MIL-125@DVMA had a severe necrosis effect on the tumors, indicating that the drugs can effectively accumulate at the tumor site to inhibit tumor growth. Compared to the significant damage of free DOX group, there was no obvious necrosis in the heart of the nanoparticle groups, which showed that the pH-sensitive DV prodrug that was encapsulated in HA-MIL-125nanoparticles can reduce the side effects of DOX.

## 4. Conclusions

In summary, HA-MIL-125 was used to co-encapsulate a pH-sensitive DV prodrug and the autophagy inhibitor 3-MA as a new nanoparticle system for the reversal of tumor MDR. The preparation, formulation evaluation, cytotoxicity and uptake, antitumor effect and pathological examination were evaluated. The results showed that HA-MIL-125@DVMA nanoparticles were successfully prepared with good size and pH-sensitive release properties. DOX released from the nanoparticle was improved by 2.43 times in the tumor microenvironment. In addition, HA-MIL-125@DVMA enhanced drug accumulation in MCF-7/ADR cells via receptor-mediated endocytosis for the effective killing of tumor cells, resulting in reversing tumor MDR. HA-MIL-125@DVMA can inhibit DOX-resistant tumor development and reduce the harmful side effect of DOX in vivo. Compared to free DOX, the inhibition rate of HA-MIL-125@DVMA increased more than 3.5 times in the resistant tumors. Therefore, by inhibiting autophagy and P-gp efflux in drug-resistant cells, the tumor-targeting HA-MIL-125@DVMA nanoparticles presented a synergistic MDR reverse effect and can be utilized as a novel pathway for tumor resistance treatment.

## Figures and Tables

**Table 1:** Drug-loading content (DC), sizes and zeta potentials of the nanoparticles.

	DC/%	Size/nm	PDI	Zeta Potential/mV
HA-MIL-125@DVMA	31.2 ± 2.1	209.4 ± 3.5	0.231 ± 0.027	−9.31 ± 0.62
HA-MIL-125@DV	30.7 ± 1.3	217.3 ± 2.8	0.326 ± 0.062	−8.73 ± 0.43
HA-MIL-125@DOX	32.1 ± 1.6	212.7 ± 1.9	0.247 ± 0.052	−8.45 ± 0.57

## Data Availability

Data are available on request from the corresponding author.
